# Fragility and Resilience: Stories of Recovering From Hip Fractures in the Oldest-Old Age

**DOI:** 10.1177/10497323231215954

**Published:** 2023-11-29

**Authors:** Bodil Tveit

**Affiliations:** 1 Faculty of Health Studies, 87368 VID Specialized University, Oslo, Norway

**Keywords:** hip fracture, oldest-old, resilience, narrative, recovery

## Abstract

This study uses a narrative approach to explore the experiences of adults in the oldest stage of old age after they suffered a hip fracture. The focus was on participants’ perceptions and descriptions of the traumatic event, the recovery process, and the impact of the fracture on their lives. The study had a longitudinal design and included interviews with 10 participants (mean age 89) who had suffered hip fractures. Up to three semi-structured interviews were conducted with each of the participants (a total of 27 interviews) over a 3-month period. The first interviews were at the hospital, the second at municipal rehabilitation facilities, and the third at the participants’ homes. The material was analysed by means of narrative analysis. The results show how the incident affected the participants’ active and meaningful lives and how they seemed to mobilise their resources and motivation to train and recover to be able to come back home and resume the life they had before the hip fracture. The study provides nuances in the understanding of how a hip fracture can impact lives in old age. The stories emphasise the resources and capacity for resilience elderly people can possess and the importance of listening to the individual life stories, situation, personal goals, and needs when planning services for elderly people recovering from a hip fracture.

## Introduction

Peter (aged 89) was taking his daily walk in his neighbourhood on a warm, late summer’s day when his eyes fell on a nice rose climbing the wall of one of the city houses. He stepped over a small hedge and approached the flower. He admired the rose and sensed its perfume before returning to the road to continue his walk. Unfortunately, while doing so, he forgot to take note of a low fence between the lane and the road, and seconds later, he found himself lying on the pavement with a distinct pain on his left side.

At that moment, Peter had joined the sad statistics of elderly adults who have suffered a hip fracture. Hip fractures have been described as the archetypical geriatric health issue (Toscan et al., 2013). Not only are they the most frequent cause of hospitalisation for older people, they are also a perfect example of a health issue that has a tremendous cost for society and huge consequences for individuals (Czerwinski, 2022; Hektoen et al., 2016). Hip fractures are often described as ‘life changing’ (Gesar et al., 2017) or even ‘life breaking’ (Asplin et al., 2021; Zidén et al., 2008) incidents. The concept of ‘biographical disruption’ has been used to understand serious illness experiences, especially when their onset involves the sudden introduction of novel and severe symptoms (Bury, 1982; Engman, 2019). The concept describes illness as not only a rupture in ‘the fabric of everyday life’ but also as a disruption to how people understand and picture their future lives (Cluley et al., 2021). In this article, attention is paid to the experiences and stories of a group of home-dwelling people in the oldest stage of old age as they experience and recover from a hip fracture.

Medical research has to a large degree concentrated on the fragility associated with hip fractures. The concept of ‘fragility fracture’ refers to so-called ‘low-energy trauma’ resulting in a fracture due to factors often associated with advanced age. Hip fracture is the most significant fragility fracture (Abd Kahar et al., 2022). The mortality rate is high and increases with age (Kristensen & Kehlet, 2018). Even after getting through surgery and the first weeks and months of recovery, there is still a high risk that one will have to say goodbye to an independent life, if one had such a life before the incident (Killington et al., 2016). The countries of Northern Europe have the highest reported incidence of hip fractures worldwide (Forsén et al., 2020; Werner et al., 2022). The reason for this is still unclear; however, explanations for it have been linked to geographical, ethnic, and lifestyle differences.

Most of the research on hip fractures has focused on the medical and caring aspects of the pre-, peri-, and post-operative phases of the injury. Studies have found that recovery is dependent on several factors, such as age, walking ability, and comorbidity (Araiza-Nava et al., 2022); that fear of falling and psychological distress are common consequences (Visschedijk et al., 2014); that infections, sores, and ulceration are hindrances for discharge (Waring et al., 2016); and, that weight loss, delirium, depression, pressure ulcers, falls, and urinary incontinence are common problems following a hip fracture (Popejoy et al., 2013).

Relatively few studies have targeted *the oldest-old patients’ experiences* after hip fracture recovery. A review and meta-synthesis published in 2016 looked at experiences of well-being and self-confidence after a hip fracture and included 29 studies from 1986 to 2014 covering all age brackets from 65 and upwards (Rasmussen & Uhrenfeldt, 2016). The results were quite mixed, spanning from experiences of adaptation, adjustment, and supportive interaction to those of worry, missing interaction, and obstacles. Another qualitative systematic review published in 2021 focused on elderly patients’ perspectives on treatment, care, and rehabilitation after a hip fracture (Abrahamsen & Nørgaard, 2021). The review included 17 studies from 2003 to 2019 covering all age brackets from 65 and upwards, and found that the experiences are quite diverse and that both health-related factors and experiences of the health care was considered important for the recovery. The study emphasized that age, pre-fracture conditions, and personal goals should be recognised as important factors in hip fracture patients’ recovery. The fact that most research of hip fractures combines all age brackets from 65+ in one group emphasises the need for firsthand accounts of the lived experiences of this oldest-old age group (van Rhyn et al., 2021).

The strain and vulnerability associated with going through a hip fracture in old age is beyond doubt, and older people are often aware of the poor outcomes (Gesar et al., 2017). However, there is also research that has pointed at the distinct resources sometimes connected to old age. Several studies have focused on the possible role of resilience in dealing with adversity and disability in late life (Brinkhof et al., 2021). Their findings showed that a higher level of resilience can somehow buffer the frailty and vulnerability that follows serious health issues. Resilience is linked to the process of adaptation when faced with trauma, threats or significant stress (American Psychological Association, 2014). A review article on the impact of resilience in older age suggested that older adults are capable of high resilience regardless of their socio-economical background, personal experience and declining health (MacLeod et al., 2016), and that the oldest-old adults aged 85 and older sometimes tend to have a greater capacity for resilience than those who are younger. This may suggest a capacity to maintain healthy adaptive patterns, which again connects resilience with longevity. William Randall (2015) suggested that there is an often overlooked capacity for resilience embedded in older people’s self-accounts. He posited that narrative resources – how we ‘story’ our lives – may feed resilience (Randall et al., 2015). Narrative openness can be looked upon as a prerequisite for developing/maintaining identity in later life (Bohlmeijer et al., 2014).

The aim of the study is to explore the experiences of adults in the oldest stage of old age on the road to recovery from hip fractures. The research questions are as follows: How did the participants picture the event of the hip fracture in their life course, how did they experience the recovery process, and how did their hip fractures affect their ongoing lives?

## Methodology

The study had a qualitative and longitudinal design anchored in phenomenological and hermeneutical research traditions focusing on people’s life worlds and life experiences (Lindseth & Norberg, 2022). The longitudinal approach (in this study, three interviews were conducted with the same participants across a time span) is used to explore and capture aspects of time and change (Audulv, 2022). The design aimed to explore trajectories and changes in people’s life experiences during the process of recovery from a hip fracture.

### Participants

The 10 persons participating in this study were recruited at a local hospital 2–4 days after each incident. The participants were all destined for further rehabilitation at a community rehabilitation centre. The inclusion criteria for the study were as follows: aged over 80 years, home dwelling before the incidence, and with cognitive capacity to consent. A research nurse at the hospital participated in the inclusion process by selecting eligible patients based on the inclusion criteria, distributing information about the study, and asking them if they would consider participating in it. If a patient was positive to the suggestion, the researcher was informed and contacted the patient at the hospital. The patients received more information, and if they confirmed their willingness to take part in the study, a consent form was completed and a short interview and arrangements for further contact were made. Ten informants (three men and seven women) agreed to participate. The number of informants was considered large enough to gain a rich material for the study purpose and at the same time manageable as a material to provide in-depth analysis. The participants were aged 82–93 years (mean 89 years) and lived in an urban area (seven alone and three with their spouses). Four of the participants did not have children (see Table 1).

**Table 1.** Participants.

Participants	Age	Social situation	First meeting at hospital	Interview at the rehab	Interview at home
John	94	Widower, three children	v	*John died at the hospital*	
Elisabeth	87	Married, two children	v	v	v
Dagny	88	Widow, two children	v	v	v
Anna	92	Widow, no children	v	v	v
Ruth	86	Widow, no children	v	v	v
Paul	93	Bachelor, no children	v	v	v
Kristin	88	Married, four children	v	*Kristin was too confused at the rehab centre to take part in further interviews*	
Lisa	82	Divorced, four children	v	v	v (phone)
Astrid	90	Widow, two children	v	v	v
Peter	89	Widower, no children	v	v	v

### Interviews

In the interviews, the participants were encouraged to recount their experiences and, during the conversations, elaborate on and go deeper into events and situations. A thematic interview guide was used but not followed strictly. It acted more as a reminder of the important issues to touch upon during the conversations. The first interview/meeting took place at the hospital when the participants were still more or less bedbound after their surgery, although they were reaching the end of their expected 5-day stay there. The short conversations (about 15-minute duration) allowed the participants to tell details about the incident leading to the hip fracture and the experiences and thoughts so far in the process.

The second interview took place at the various community rehabilitation centres to which the participants had been allocated for an average of 3 weeks around-the-clock rehabilitation. The interviews (about 1-hour duration) focused on experiences of the rehabilitation process as well as the participants thougths about going back to their homes. The interviews were conducted in separate rooms, since the participants often shared rooms with a fellow patient; however, in two cases, the participants had single rooms, and their interviews took place in the respective rooms.

The third meetings were arranged to take place in the participants’ homes 10–12 weeks after their hip fractures. One of the informants did not want me to visit her home, saying that she felt that it was too messy, so an agreement was made to conduct the interview over the phone. The others received me into their homes often with a cup of coffee and a slice of cake in their living rooms and were well prepared for a longer discussion. The interviews (about 1½-hour duration) focused on experiences from the whole process, the daily life after coming home as well as prospects and thoughts about their further life.

The longitudinal approach with three face-to-face meetings allowed for an individualised approach whereby the previous information provided by the informants became more than background information and guided the process further as the recollections were followed up. The participants accepted that the interviews would be recorded.

### Narrative Analysis

Shortly after the interviews, I transcribed them verbatim. Supplementing the interviews, shorter field notes were taken during the more informal talks at the hospital and during the informal exchanges on the phone before and after interviews.

It was when I started analysing the material that the narrative form and content of the material struck me. The interviews were saturated with narrative elements and contained as a whole sequence of an overall story. The orientation, plot, and complicating event, followed by some form of resolution and evaluation were discernible in almost all the interviews. The longitudinal approach, with three different meeting points with the participants along the pathway, underscored the story line and stages.

Narrative analysis is not one uniform or standardised analytic tradition within qualitative research. There are different ways of doing it, and narrative analysis is often pictured as flexible and with room for intuition (Gubrium & Holstein, 2009; Riessman, 2008). My analysis of the participants’ stories started with reading all the interviews separately and trying to capture the individual stories in the material. There were different stories and various ways to tell them. After working with the individual stories, I focused on the connections between the individual stories and the overall story I could read out of the entire material. Through combining elements from all the stories, I tried to grasp and retell the overall story in the material as a whole by zooming in important elements of three different narrative sequences. When analysing the material, some conceptual tools provided resources for me to be able to see, understand, and discuss the individual stories and the overall story I read out of the material as a whole. I have already mentioned the concepts of biographical disruption (Bury, 1982) and narrative resilience (Randall et al., 2015) which both came up in the process of analysing. Arthur Frank’s (2013) description of three different types of *illness narratives* in his book *The Wounded Storyteller* was also useful in understanding the stories and realising that these types are not mutually exclusive.

I asked several analytic questions to the material:

• How are significant events narrated?
• How do they narrate their new daily life?
• How do the participants picture the health care they are offered? What are they asking for?
• What is at stake in the participants’ stories?

In the process, I felt a strong commitment to be true to the stories told to me. However, I am aware that my story based on their stories is somehow depictive, connective, and selective (Lindemann, 2017), in the sense that I depict actions to report, link them together, and choose from among several possible details. I am also aware that this process is interpretative, as it adds to the overall meaning of the story, even if all the events and single points are carefully and accurately reported in the way that they appear in the material (Frank, 2010). Riessman (2008) argues that narrative truths are always incomplete and partial. It is important to note that some of the participants were also practiced storytellers and seemed to enjoy talking about their experiences; they sometimes appeared to be quite aware of what story they wanted to communicate.

### Ethical Considerations

The study was registered and approved by the Norwegian Centre for Research Data (no. 47863) and by the participating hospital’s ethical research committee before recruiting the participants. The recruitment process was performed and carefully discussed with a colleague and with the research nurse at the hospital. I was aware that the patients would be in a stressful and vulnerable situation, and I wanted to make sure that they had understood the project and the implications of agreeing to take part in it without feeling any pressure to do so (Biros, 2018). In addition to receiving oral information from the research nurse, they received written information about the project. Based on this information, they decided whether or not they would like to meet the researcher (me). Two patients who fitted to the inclusion criteria declined after receiving the information, but those who said yes to meeting the researcher agreed to participate and signed the written consent form during the meeting with me at the hospital. The participants were contacted by phone prior to the second and third interviews, and they confirmed orally that they would like to take part in the next interview and agreed to meet me. The material has been anonymised and any identifying information removed from the data without changing the meaning. Since I had met the participants three times, I was able to discuss some of the early findings with them to validate the interpretations (Slettebø, 2021). In the final interview, I offered them the opportunity to see the transcripts from previous interviews, with one of the participants wishing to read through her interviews; she had no objections to the transcript.

## Results

The participants of this study were at the outset 10 patients recruited during their stay at the hospital. However, going through a hip fracture is akin to going through a physical and mental crisis. There are risks involved. John (94), one of the participants, was a smiling, white haired widower. When I visited him at the hospital to make an appointment for the first interview, he received me with warmth and positivity. While lying on his hospital bed, he confirmed that he wanted to participate in the study. His daughter was at his bedside, and he was expecting a visit from his grandson when I was there, so we just had a brief conversation before we said goodbye and agreed that I should call him at the community rehabilitation centre, where he had been told he would be transferred over the weekend. The day after, on the fourth day after his surgery, he died due to a pulmonary embolism.

Another example of a possible complication of a hip fracture is the story of Kristin (88). She became somehow confused, almost delirious, during her hospital stay, although she seemed coherent when I visited her, and she confirmed that she wanted to participate in the study. However, when we met again, at the community rehabilitation ward, she was noticeably agitated and struggled to answer my questions and participate in a meaningful conversation. She was positive and kept saying how fantastic everything was, how good she felt, and that she was soon going home to take up her household duties. Her state made it difficult to reschedule an appointment with her for a follow-up interview.

The results are based on the conversations I had with all ten informants, even though it was only possible to follow eight of them throughout the process. I have chosen to present the findings focusing on three narrative sequences, starting with the incident that caused the fracture (the complicating event) and continuing with the participants’ experiences during the rehabilitation process at the hospital and rehabilitation centres and finally concentrating on their stories after they had returned home. The presentation is based on analysis of all the interviews; however, I am going deeper into some of the participants’ stories to illustrate key points.

### Caught in the Midst of Everyday Life

Like Peter, in the introduction, most of the participants recalled how their hip fractures had hit them unexpectedly as a sudden event that interrupted their relatively stable and active lives. Some informants had been inside their homes when the incident happened. Kristin (88) slipped on a slippery bathroom floor; Elisabeth (87) was watching the news on television when her leg ‘fell asleep’ and gave way beneath her as she got up from the chair. Several of the informants were *outside* their homes when the incident leading to the fall happened. Astrid (89) knew that she was taking a risk when she hurried along an icy road to be in time for the train to take her to a hairdressing appointment. Ruth (86) had been out for a little walk shopping, with her rucksack on her back, when she suddenly tipped over when she ascended the pavement after crossing the road. Paul (93) was in a shop buying groceries when he bent down to pick up a lemon that had fallen to the floor, leading to a sudden fall. Anna (92) had a more extraordinary, annoying experience leading to her fall. She said:

It was a dog! I was out having a walk in a park close to where I live. It was completely wild, and the owner should have kept it in a lead, but then it attacked me, and I fell. Then it [the hip fracture] happened.

A further insight into Anna’s and Paul’s stories can serve as examples of the everyday lives older people may be leading prior to a hip fracture.

Anna (92), who has been widowed 4 years previously, has moved from the villa she shared with her husband to a new, sixth-floor flat in a building with a lift close to the city centre to live more practically in her older age. She has no children but lives in the same neighbourhood as her younger sister (86), with whom she is very close. The two of them have enjoyed the city life together, going to art exhibitions and theatre performances, having lunch in cafés and so on. She used to work in a publisher’s office and has for many years lived abroad with her husband, who was an international businessperson. She is very fond of reading and can speak several languages fluently. Prior to the incident, she described herself as an active and fit woman, although she was very aware of her age, saying, “Of course, I could somehow feel my age, but I would go out for a walk every day. Only in the few last months before the incident had I felt a bit unsteady, but I managed well.” The previous summer, she had taken the initiative of visiting a nursing home in her neighbourhood, just to get an idea of what it looked like. She was surprised to see that most of the rooms did not have a separate bathroom, saying, “It was too bad. I didn’t want to end up there. But when you are 92, you must be prepared that this might happen.”

Paul (93), a bachelor all his life, had held a responsible position in an industrial company. After his parents died, he continued to live with his younger sister in the apartment they inherited. They were very close. The sister died a year ago, and it was a hard blow for him. He is not sure who his closest relative or next of kin is now – possibly his cousin’s children. Before the hip fracture, he had been doing well at home, but he had felt more tired, and his back had started to bother him. Things have gradually become more difficult, and his walking function had deteriorated. Before the hip fracture, he received help from home care services, who provided supervision and assistance with his support stockings. In addition, a private cleaner visited every second week. He was frequently in contact with his neighbours: “I am lucky that I have very nice neighbours.” Paul is very interested in food and enjoyed cooking his own dinners. He is not very fond of precooked microwave food and thought it is important to buy high-quality ingredients. He would usually take walks by himself to a store a few hundred metres down the street with the help of a walker. “Unfortunately, the local fish and game store closed recently, so now I have to go a little further to get to a store with a deli counter,” he said.

Even though many of his friends are now dead, Paul still has some friends who he would meet regularly and with whom he plays bridge. “The average age of the group is 93,” he said. He used to be a passionate photographer and a stamp collector, but he has put those interests aside in recent years.

#### Reflection

Anna’s and Paul’s stories picture life situations that are quite typical of those experienced by several participants of this study. Despite their old age, they seem to engage vigorously in daily activities and lead meaningful lives. The incidents that led to their hip fractures took place rather suddenly and unexpectedly while they were carrying out everyday activities. However, in several stories, the incidents seemed to occur in the wake of slightly declining health conditions. Their falls – Kristin’s in the bathroom, Elisabeth’s in her living room and Paul’s while bending down in the shop – may all indicate experiences of bodies as ‘compromising safety’ or bodies that cannot be trusted to the same extent as previously (van Rhyn et al., 2021). However, the overall impression is that when their falls occurred, the participants were still maintaining their independence and important qualities of life.

### At the Hospital and Community Rehabilitation Facilities: Motivation and Frustration

Hip fractures disrupt everything in everyday life. Suddenly the participants found themselves in an ambulance; they were transported to the hospital, examined and tested, and within 24 hours, they went for surgery. The participants in this study were included in a fast-track programme at the hospital, implying a 5-day standard pathway before transfer to a rehabilitation unit. However, mobilisation and the rehabilitation process started at the hospital. Several mentioned that they had been up and walking very early after the surgery. Anna is one of them. She praises the hospital and the stay there. She was out walking the day after surgery and recovered quickly, she thinks, during the first few days.

Astrid (89) had some complications after the hip fracture, including an infection, a blood clot in her leg and a minor heart attack during her hospital stay, causing her to spend longer time in hospital. However, some of the participants had problems remembering much from the rehabilitation and training they were offered at the hospital. “Huh, my time at the hospital has become so strange, I can’t remember it. It was so messy,” said Dagny, 88.

Paul had a clear memory of at least some of the training at the hospital, saying:

I had a physiotherapist there. A tough type. He was very eager to make me able to do things, such as lifting my leg and get into the bed by lifting the injured leg with the good leg. Normally, at that time, the pain was from 1 to 4 (scale from 1 to 10), but once, when he pushed me, it was 10!

The participants included in my study were all offered a 3-week stay in a community rehabilitation centre immediately after discharge from hospital. There were a few different centres, and the participants were each sent to one depending on where there were available beds. Their experiences of the stay and training offered at the community centres were mixed.

Lisa (82) pictures the rehabilitation she has been offered as very good. Mostly, it is offered by physiotherapists and consists of walking in stairs and just learning to walk correctly with a walker and later with crutches. The training is twice a day. She manages her personal care and can walk on her own with a rollator (a wheeled walked) now. She is sharing room with a woman of her own age, and they have really tuned in and are joking a lot together. She is laughing a lot during the interview.

Anna (93) was somewhat unfortunate, having arrived at the rehabilitation during a period filled with public holidays. After 3 weeks, she was about to be sent home, but she refused: “I told them that I had just received a few hours of physiotherapy and I am here for training.” She was given another week there and some more hours of physiotherapy, but most of the time, she would practice trying to walk correctly on her own with crutches, rather than using a walker. The physiotherapist gave her four exercises to be done four times a day: “To bob with your legs, you know, and pull up your butt, tighten your thigh muscles and lift up your legs.” Anna emphasises the need to work on her own: “You have to take control yourself!” She has lost weight and appetite but tries her best to eat as much as she could and take the nutritional drinks offered to her.

Paul (93) describes the training offered as “fair enough, but not great.” Like for the others, the main training he is offered consists of walking exercises both on a flat floor and on stairs. “It’s only 30 minutes per day, except on weekends.” He is struggling to get dressed without help, especially putting on his shoes and socks. He still has pain in his hip and back, for which he is offered medication. Like Anna and Astrid, he has lost his appetite. Still, he tries to eat as much as he could, saying, “The food I make myself at home is much better!”

Astrid feels that all her complications after the hip fracture have set her back and influences the rehabilitation process. She is exhausted and struggles to get through her days. She said:

There’s a lot going on here, and then you must get up, get dressed and then go out for breakfast; so, after breakfast I often lie in bed for a while. And when there is food, I have no appetite.

She knows that she needs to train on her own in addition to the lessons she has been offered with the physiotherapist. The physiotherapist gave her a booklet with exercises in it, but she has been tired and finds most of the exercises too difficult.

Elisabeth has a particularly negative experience. The place she had hoped to go to was full, and she was offered a place at another institution, a so-called health house, while she waited. When I meet her at the health house, she is lying on her bed, deeply frustrated. She has hardly seen a physiotherapist and feels that the rehabilitation process has not really started. She has called the municipality, but with no result so far and says:

I cannot accept this. I feel abandoned! I didn’t expect much, really; I was just going to stay here for a couple of days until they found a place available, but now it’s been almost 3 weeks. Maybe they think that 87 is too old and that it’s the right time to go home and die?

Elisabeth is not blaming the staff, and she feels that the nurses are running around like crazy: “They are angels, all of them; they are so sweet and kind and supportive, but they don’t have enough time. They are running around all the time.”

She tries to perform exercises herself:

“I need stronger muscles, and I need to train them. I am not used to training at home. I am lazy. But now I am motivated,” she says. “I must get up on my feet. I have no intention of spending the rest of my life in a wheelchair. […] How long am I going to lie here stored away on a shelf? I am now so tired that I may as well just go home. But it would have been better to get a place with some real rehabilitation. I need bodybuilding!”

#### Reflection

Most of the stories indicate a strong sense of motivation. Many participants picture themselves as highly motivated to perform exercises, get up on their feet, practise and train. They understand clearly that a lot is at stake; their future lives and independence at home are at risk. The rehabilitation programme seems to offer some support. However, the participants have mixed experiences, and Elisabeth’s unique experience of being outside the mainstream programme and being ‘stored away’ underlines the importance of ensuring proper rehabilitation services. Many participants encounter obstacles related to their weight loss, lack of appetite, pain and psychological distress. These are common problems related to hip fractures (Araiza-Nava et al., 2022; Langford et al., 2018). However, the participants seem to be somewhat left alone in struggling with these issues. It is interesting to hear Anna expressing her view that she herself has to take control and negotiate a longer stay than is offered in the standardised package. Unlike Anna, several participants seem to settle for what they are offered without demanding much more.

### Back at Home: Wishing for a Normal Life

The possibility and desire to go back to their homes seem to be an important motivation for the participants to try to make the most of the rehabilitation process and regain functionality and strength. A longing for home and the joy of going home is a common feature in all the individual stories. Their homes varied in size and shape from larger upgraded apartments with thick carpets and art on the wall to smaller flats with worn furniture, family pictures, and old-fashioned interiors.

Peter (89) is enjoying returning to his life at home after weeks away at hospital and his subsequent rehabilitation. He lives alone in his central, two-room apartment that his late wife inherited many years ago from her aunts. Most of the furniture is from the aunts, and he likes it that way. After 40 years of working on the floor in an industrial firm, he feels that he is in a comfortable position in terms of his economical and material resources. Being back at home, he has continued his routine of doing exercises every morning. He shows me how he does push-ups and squats. He used to play football when he was young, and he still feels that he is quite fit and strong, despite having become a little stiffer and feeling somewhat rusty. He has resumed taking short daily walks in the neighbourhood to get some fresh air, do his shopping, and watch the life around him. He has a sister in another part of the town whom he meets regularly.

Dagny (88), who is a widow with a family encompassing two children, three grandchildren, and one great grandchild, is very pleased to have returned to her old life. She lives alone in her own flat near the city centre. Even though she might have needed some assistance, she has rejected the offer of having home help and wants to try to manage on her own. She is tired of having people coming and going at erratic times. “There were too many people at the door,” she says. During the interview, she receives a visit from her next-door neighbour, an aged former professor. They are old friends who have re-acquainted themselves by chance. They quite often enjoy each other’s company, regularly eating dinner together, and he is the one who shops for her groceries.

Paul (93) has returned to the flat he grew up in and shared with his sister until she had died, some years ago. He appreciates being back, saying:

What’s important for me is to be able to walk around in my home and be able to go shopping for groceries. I have my daily routine. One day, I wash my clothes; twice a week, I water my plants; and I watch TV and prepare my meals.

He still has a car, and the previous week, he took it out of the garage to try it out. “I had one of my relatives to help me, of course. We drew up to a forest nearby. It went well,” he says. Paul regrets not being able to use the car more often*.* “It is too difficult. But it is parked right outside here, on the street, and now it works perfectly after I got the battery changed,” he said. Paul’s dream is to drive to his cabin some hours outside the city. “It has no purpose when I can’t walk properly, since the terrain is hilly,” he said*.* He has half-arranged an appointment to go there for a daytrip the following week with one of the daughters of his cousin. “The cabin is just there, empty. Nobody uses it,” he says. Paul admits that he no longer feels healthy. “Without support and help, things would go badly. But at my age, what would you expect?” he says. He has tried to ask the doctor for his prognosis and prospects, but the answers were vague. When pondering about the future, his thoughts would turn to the people he has lost in recent years. “It is sad to lose people around you. I just have a couple of friends left of my own age,” he says. Paul uses the phone frequently to keep in contact with his few remaining friends.

Elisabeth returned to the flat she shares with her husband and tries her best to manage. Regaining normal function has taken time. “In the beginning, I didn’t know how to get through the night,” she says. “But I have gradually managed to find a way to curl up into a better position.” Her husband and their daughter, living close by, would help her, and she also receives some assistance from the municipality’s home care services each day. She hopes that her leg would improve and that she would become stronger. “When I get strong enough and able to walk properly, hopefully I will manage on my own; that’s what occupies my mind. And maybe, I will get back to having something like the life I had before,” she says.

Astrid’s story is special. She felt exhausted when she came home from rehabilitation. She lives alone in a big flat, where she walks around with a walker and serves me coffee and homemade cake. “I should have moved to somewhere smaller when my husband died, but I felt safe here at the time,” she says*.* The physiotherapist gave her some exercises that she was supposed to do on her own at home. She has not yet looked at them and feels that she has no capacity to do them. People from the municipality’s home care service have visited her, but Astrid became very frustrated and almost angry after the visit. She felt that she was being pushed into setting goals and doing exercises, among other things. “Maybe I am vulnerable. I guess I am,” she says. “But I have been through a lot over many years. They do not understand that.” Astrid feels that she has the right just to be at home and to find her own way without being pressured. “If I can make it at home, it’s up to me, isn’t it? I am not going to work; I am not going to accomplish anything big. I am just going to be Astrid and stay at home and manage,” she adds.

Astrid expresses mixed feelings during the interview. She feels happy about being at home, and she seems somehow optimistic about her recovery. She says: “I have been worried that I will end up using the rollator for many years, but my brother said: ‘Don’t worry; when the summer comes, you will walk without it.’” She hopes that she would be able to go to the family cabin as usual in the summer. But when thinking about it, her mind turns to a less optimistic picture:

“I am certainly going there one more time, in an urn,” she says. “And then, I will be scattered on the land we own, just like my husband. I guess you will see my name in an obituary in a couple of years’ time. I hope I stay here until they carry me out.”

#### Reflection

Although the participants’ experiences vary, being at home seems to provide a sense of life quality for all of them. However, returning home has not necessarily been easy for them. For some, their reduced functions adversely affect their daily lives. They struggle with their abilities to perform normal activities, such as bathing, shopping, cleaning or even getting dressed in the morning. Many of them are grateful for receiving home support, which helps them cope with everyday living. Despite these problems, there is a clear tendency in the stories: The participants still have interests, needs, wishes, and hopes for their days to come. Paul’s example is striking when he talks about repairing his car hoping for another trip to his cabin, as is his mention of cooking his own dinner rather than just accepting the ready-made food that he finds tasteless. Anna is hoping and looking forward to resuming her walks with her sister and visits to the galleries. Dagny is yearning for privacy to maintain her life with her close friend.

In most of the participants’ stories, there is a strong desire and hope to return to life as it had been prior to the incident and to be able to enjoy life. At the same time, there seem to be an awareness of how precious each day is and insecurity about the future. The concept ‘suspense’ refers to a tension between different possible outcomes – those to be hoped for and those to be feared (Frank, 2010). Going through a hip fracture not only temporarily reduces the health of the participants but there is also a risk that the setbacks could become permanent and even lead to a further declining health situation.

## Discussion

This article is based on an analysis of interviews with elderly patients on the road to recovery after sustaining hip fractures. In this study, the aim was to explore the experiences of adults at the oldest stage of old age and on the road to recovery from hip fractures. The research questions are as follows: How did the participants picture the hip fracture incidents in their life courses? How did they experience the recovery process? How did their hip fractures affect their ongoing lives?

### Illness Narratives and Biographical Disruption?

Using Arthur Frank’s (2013) typology of *illness narratives* can be a useful lens to understand the stories. However, rather than being either/or, the stories appear as combining elements of all three types. Most of the individual accounts can be read as *restitution stories* whose different sequences point to the steps in the process of rehabilitation towards regaining the participants’ (walking) function, as well as managing at home. However, the *chaos* is visible in many stories. The fall itself occurred abruptly and shockingly, and the hours and days after the fracture are pictured as chaotic and stressful. Many participants struggled to remember their time at the hospital when I met them at rehabilitation facilities. For some of them, most likely for Kristin and Astrid, the chaos seems to continue and even be more dominant. There is also a quite noticeable *quest* in many of the stories: Will I manage at home? Will I reach my goal? How long will it last? Or as Astrid puts it: “I hope I stay here until they carry me out.” There is sometimes an unspoken and at other times an articulated awareness in the stories about the fading future prospects and the unavoidable end to it all.

The stories they tell somehow align with the concept of biographical disruption (Bury, 1982). According to Bury (1982), there are three facets of biographical disruption when experiencing severe illness: (1) a disruption of the taken-for-granted assumptions and behaviours, (2) a fundamental rethinking of the self-concept and (3) a mobilisation of novel resources. In the stories in my study, the participants’ self-concepts seem to stay quite stable in the sense that most of them seem to integrate the new situation quite rapidly into their understanding of themselves and their lives. Experiences of biographical disruption when going through severe illness vary with age, and older people do not necessarily face a distinct rupture in identity. Engman (2019) builds on Bury’s work in her phenomenological study of understanding severe illness. She emphasises the importance of former experiences of physical impairment as part of an embodied sense of the world and discusses how rupture and continuity go together in many cases. She opposes the thinking that either the rupture manifests itself or it does not (Engman, 2019). This is in line with my study’s results, where the participants seem to mobilise the resources they possess to avoid disruption and whenever possible strive for continuity.

### Narrative Resilience and Counter-Stories

The resources the participants possess can be labelled as their capacity for resilience. Previous research has suggested that older people can be capable of great resilience, which seems to buffer their frailty and vulnerability when going through serious health issues (MacLeod et al., 2016). The fact that my study’s participants had an average age of almost 90 years is interesting. Some scholars have suggested that to some extent, resilience can be connected to longevity and may be shaped by having experienced stressors and hardship in the course of a long life (Luthar & Barkin, 2012).

According to Frank (2010), telling a story and creating an account of what happened and how to deal with it can serve the function of ‘holding one’s own’, in the sense of maintaining self-identity in conditions where it is threatened. Keeping this in mind, it is possible to perceive the stories my study’s participants told me as resources for resilience that worked for the people involved. Stories not only represent an existing capacity but also shape, strengthen and nurture the storytellers’ ability to cope with their conditions. William Randall (2015) pointed to older people’s particular narrative capacity and how self-accounts can be used to ‘feed resilience’. Some of the participants of my study likely found resources when focusing on possibilities and hope when they pictured their life situations and future prospects.

Hilde Lindemann (2020) utilised the concept of counter-stories to describe a sort of resistance that individuals or groups can put up if they find that the *master narratives* that society offers them and the cultural plotlines or identities that dominant groups construct for them are unsatisfying. The counter-story is a strategy of resistance that allows identity to be narratively repaired. In line with this, my study’s participants provide features and elements in contrast to the overall impression from the ‘fragility’ stories in the research on hip fractures, as presented in the background section. The impression of another story, a counter-story, is strong and prominent.

Although a sense of hopefulness may be important to underpin and develop resilience and serve as a counter-story that can be a resource for the participants (i.e. in their recovery after their hip fractures), there is always a chance that counter-stories can backfire (Lindemann, 2017). In cases where the set goals and wishes do not meet real possibilities, it may contribute to suffering, as well as constant and sometimes unhealthy feelings of inadequacy and falling short of their self-expectations.

### What Is at Stake?

An important finding is that the stories point to inadequacies of the health care system. In particular, the rehabilitation offered at the various facilities seems to fall short and, in some ways, fails to meet the needs of the participants. Quite a few of the participants suffer from complications after their hip fractures, such as loss of appetite and weight, as well as pain problems, which are all common issues. Others have more severe complications; for example, Astrid suffered infection, thrombosis and a minor heart attack during her hospital stay. These seem to set her back and make her stay at the rehabilitation facility and the return home much more difficult. The services that she receives seem to be far from what she thinks she needs, and her communication with the health care provider appears to have broken down.

Elisabeth’s description of nurses running off their feet and her desperate feeling of being stored away at the health house, without receiving the training that she needs, again lead to her question of whether the staff perceive people of her age as dying. Her situation points to the problems with the overburdened health care system and the suffering it may cause to patients. Exercise is considered the key for regaining mobility after a hip fracture (Vestøl et al., 2021). The stories’ overall message is that a lot is at stake for the participants of this study. Being able to come back to their homes, sleeping in their own beds, cooking nice food, talking to their neighbours and resuming daily routines are all of immense importance to them. Being in familiar surroundings can help people maintain their self-conception and self-identity (Lindeman, 2017). Hence, undergoing proper rehabilitation and receiving the needed support is essential to be able to achieve this. Most of them are also highly motivated to put much effort into the rehabilitation exercises and activities, knowing that this is important.

Astrid’s resistance against the coping team and insistence on not following instructions and not being part of the training programme are notable. However, she points to an important aspect of being at home, namely her autonomy in choosing her own lifestyle. Previous studies have emphasised autonomy as an important success criterion in hip fracture patients’ transition from rehabilitation to their return home (e.g. Sims-Gould et al., 2012). For Astrid, this means taking responsibility for her own life and not being under pressure from outsiders, even if they are trying to help her. She wants to live her life and cope with her life in her own way.

## Conclusion

This study’s results provide further insights into how people at the latest stage of old age experience going through and recovering from hip fractures. The stories add nuances to the image of elderly people after hip fractures, often pictured as fragile, vulnerable and weak. The study pictures old persons disposing a lot of resources in their process of recovery and provides insights into what everyday life can be like at the oldest age and how people at such an advanced stage of life can deal with adversity. The study’s results underline the importance of assuring adequate rehabilitation services while considering the resources people may possess. It emphasises the importance of listening to the individual life stories, wishes and dreams, and their needs for support and assistance to be able to resume the life they want and realise some of their potentials. It is significant to recognise the threat that a hip fracture may pose to a person’s life and identity in the latest stage of old age, as well as the possibilities to recover and maintain important aspects of life after a hip fracture.
